# A comparative study of Arabic syntactic analyzers

**DOI:** 10.3389/frai.2025.1638743

**Published:** 2025-08-26

**Authors:** Omar Saadiyeh, Alaaeddine Ramadan, Mohammad Hajjar, Gilles Bernard

**Affiliations:** ^1^Paragraphe Research Lab, University of Paris VIII, Paris, France; ^2^College of Engineering and Computing, American University of Bahrain, Riffa, Bahrain; ^3^Faculty of Technology, Lebanese University, Saida, Lebanon

**Keywords:** Arabic NLP, Arabic treebank, syntactic analysis, rule-based parsing, statistical parsing, hybrid parsing, neural parsing, transformer models

## Abstract

Syntactic analysis stands at the heart of Natural Language Processing (NLP), serving as the cornerstone upon which deeper linguistic understanding is built—particularly for morphologically complex languages such as Arabic. This paper delivers a comprehensive comparative study of contemporary syntactic analyzers designed explicitly for Arabic, dissecting the strengths and limitations of rule-based, statistical, machine learning, and hybrid methodologies, and recent neural network and transformer-based models. Given Arabic's intricate morphological structure and rich syntactic variation, accurately capturing syntactic relationships poses a significant challenge. To address this complexity, our study meticulously evaluates existing algorithms, highlighting advancements, performance gaps, and practical trade-offs. In addition, recognizing that robust syntactic parsing is anchored in high-quality annotated datasets, we provide a thorough overview of available Arabic treebanks and annotated corpora, emphasizing their critical role and contribution to syntactic parsing advancements. By synthesizing current efforts in the domain, this comparative analysis not only offers clarity on the state-of-the-art but also guides future research directions. Ultimately, our work seeks to empower NLP practitioners and researchers with nuanced insights, enabling more informed choices in the development of powerful, accurate, and linguistically insightful Arabic syntactic analyzers.

## 1 Introduction

Arabic is a Semitic language characterized by complex morphology, rich inflectional patterns, and flexible syntactic structures, posing significant challenges to natural language processing (NLP). Syntactic analysis, commonly referred to as parsing, is a critical step in NLP tasks such as machine translation, information retrieval, and sentiment analysis. Parsing Arabic, however, is particularly challenging due to linguistic phenomena such as diglossia, morphological ambiguity, and relatively free word order (Habash, 2010). Numerous parsing approaches have been proposed for Arabic, ranging from traditional rule-based systems to modern statistical and machine learning-based parsers. Early rule-based systems, primarily grounded in classical Arabic grammar rules, provided foundational insights but were limited by their scalability and adaptability (Othman et al., 2003). The advent of annotated corpora such as the Penn Arabic Treebank (PATB) facilitated data-driven methodologies, leading to significant advancements, including probabilistic context-free grammars (PCFGs), support vector machines (SVMs), and more recently, deep learning models utilizing contextualized word embeddings (Taji et al., 2017).

This paper provides a comprehensive survey of state-of-the-art Arabic syntactic analyzers developed in recent years. It systematically discusses key syntactic parsing approaches, exploring both rule-based and data-driven paradigms. Further, the paper evaluates prominent Arabic syntax treebanks and related resources that have enabled significant progress in parser development. Subsequently, we compare the performance of existing syntactic analyzers across various linguistic domains and applications. Finally, the study addresses ongoing challenges and limitations within the field, outlining avenues for future research.

## 2 Related work

Most existing review papers on Arabic syntactic parsing either broadly cover general NLP tasks or have become outdated in their specific analyses of syntactic parsing for Arabic. Dedicated comparative studies with a strict syntactic orientation remain scarce, and those available often overlook recent datasets or state-of-the-art parsing techniques.

Zaki et al. (2016) conducted one of the earlier comprehensive surveys focusing exclusively on Arabic syntactic parsers developed up to 2016. They categorize the parsers based on methodological approaches—rule-based, statistical, and hybrid—and clearly outline their advantages and limitations. Despite the breadth of this work, it now lacks coverage of subsequent developments in annotated datasets and parsing methodologies introduced post-2016. A more recent comparative study by Aqel et al. (2019) addressed advancements in Arabic parsing systems, highlighting their strengths and limitations, and providing suggestions to mitigate common parsing challenges. Although informative and relatively current, this work similarly falls short in referencing the latest syntactic annotation schemes and updated parsing datasets that have emerged after its publication.

Recent surveys addressing broader linguistic contexts have also appeared, such as those by Hamed et al. (2025), examining code-switched Arabic NLP, and Xu et al. (2025), exploring multilingual large language models. While valuable, these studies primarily focus on multilingual and cross-lingual scenarios and do not specifically target syntactic parsing of Arabic, highlighting a clear gap in the literature for a dedicated, syntax-focused comparative study for Arabic.

In summary, the literature reflects a notable scarcity of recent and specialized comparative studies that focus explicitly on Arabic syntactic parsing. The present study addresses this gap by offering a comprehensive and current analysis specifically targeted at syntactic parsers for Arabic, incorporating insights from recent developments and datasets.

To better contextualize the reviewed work, we briefly outline the fundamental concepts and methodologies in syntactic analysis. Syntactic analysis, or parsing, refers to the process of analyzing sentences by identifying their syntactic structure according to a set of grammatical rules. This task is fundamental in natural language processing (NLP) and computational linguistics, as it helps in understanding sentence structure and meaning. In the context of Arabic, syntactic analysis can be approached in several ways, each offering distinct advantages depending on the complexity and formality of the grammar involved.

### 2.1 Approaches to syntax analysis

Syntactic analysis can be approached using two primary methods:

- Top-Down Parsing: This method starts with the entire sentence and breaks it into smaller parts (constituents) using grammar rules. These parts are further divided until you reach individual words. This strategy works well with grammars that focus on sentence structure (Aho et al., 2006).
- Bottom-Up Parsing: This method begins with the words in the sentence, assigning each a grammatical label. These labels are then combined to form higher-level structures (like phrases) until the full sentence structure is built. This method works with many types of grammar (Aho et al., 2006).

### 2.2 Available parsing algorithms

The selection of parsing algorithms is critical to the efficiency and effectiveness of syntactic analysis. Two prominent algorithms are:

- Cocke-Younger-Kasami Algorithm: A fast, table-based parsing method for context-free grammars, especially effective when the grammar is in Chomsky Normal Form (Brandt and Walter, 2001).
- Earleys Algorithm: A flexible algorithm that works with both normalized and non-normalized context-free grammars (Tendeau, 1997).

### 2.3 Parsing techniques

Several approaches to syntactic analysis in Arabic focus on different methods and techniques, including:

- Rule-based approach: which uses a well-defined formal grammar based on the knowledge of linguists on the language concerned;
- Statistical approaches: which uses machine learning techniques to create grammar rules from a corpus annotated (TreeBank), then analyzes the sentences using these rules;
- Hybrid approach: which uses both a predefined grammar and a statistical module (for example a disambiguation module) allowing to improve the results and to resolve the ambiguities.

### 2.4 Depth of parsing

In syntactic analysis, the term “depth of parsing” refers to the extent and precision of syntactic information extracted from a given sentence. This concept plays a critical role in shaping the goals and applications of parsing systems, especially for morphologically rich and structurally flexible languages such as Arabic. Generally, parsing approaches fall into two broad categories based on depth: deep parsing and shallow parsing.

- **Deep parsing:** Deep parsing involves generating a full syntactic structure for a sentence, capturing the complete grammatical relationships among words and phrases. This typically results in hierarchical representations like constituency trees or dependency graphs, which identify syntactic roles such as subjects, objects, and modifiers. For Arabic, deep parsers often rely on resources like the Penn Arabic Treebank and are capable of handling sophisticated linguistic features, albeit with significant computational cost (Habash, 2010; Taji et al., 2017). These parsers are valuable for tasks requiring nuanced understanding of sentence structure, such as machine translation and semantic analysis.
- **Shallow parsing:** Also known as chunking, shallow parsing focuses on identifying the main syntactic units within a sentence, such as noun phrases or verb groups, without delving into their internal grammatical structure or hierarchical organization. This approach is generally faster and more robust, particularly in noisy or resource-scarce settings. In Arabic NLP, shallow parsing is often used in applications like named entity recognition and basic information extraction, where full parsing is unnecessary (Shaalan and Khaled, 2010).

Each method presents advantages depending on the use case. Deep parsing provides comprehensive syntactic insight but demands more processing power and annotated data. Shallow parsing offers efficiency and adaptability, especially for preliminary or large-scale language tasks. In practice, hybrid models that combine both levels of analysis are becoming increasingly common in Arabic syntactic processing.

## 3 Arabic syntax treebanks and resources

The development of Arabic syntactic parsers relies heavily on annotated treebanks, which provide valuable resources for training and evaluating parsers. Notable Arabic treebanks include:

**Penn Arabic Treebank (PATB)** employs a statistical approach for annotating Modern Standard Arabic, focusing on structural morphology and syntactic analysis. It includes comprehensive annotations for parts of speech (POS), morphology, gloss, and syntactic trees. The corpus consists of 599 articles from the Lebanese newspaper *An Nahar*, totaling 402,291 word tokens. The annotations, following the Penn Treebank guidelines, are used for syntactic parsing and language modeling. Evaluation results across multiple versions demonstrate high accuracy, with more than 99% of tokens correctly tagged for POS and morphological analysis, ensuring robust reliability for linguistic and computational applications (Maamouri et al., 2004, 2005).

**Prague Arabic Dependency Treebank (PADT)** is grounded in a theoretical approach inspired by the Functional Generative Description framework and the Prague Dependency Treebank. It includes over 113,500 tokens with detailed syntactic and morphological annotations. This treebank is designed to aid dependency parsing and has been utilized in the CoNLL shared tasks, showcasing its utility in parsing experiments. The dataset covers 212,500 words, with a strong focus on syntactic dependencies. Its evaluation results highlight the accuracy of dependency relations, supporting the treebank's role in both theoretical and practical parsing tasks (Hajič et al., 2004, 2006).

**Columbia Arabic Treebank (CATiB)** adopts a simplified dependency-based approach that emphasizes annotation speed and efficiency. It provides syntactic analyses, including over 1 million tokens, with 841,000 words and 31,319 trees from newswire feeds and other sources. CATiB uses a reduced set of syntactic labels compared to PATB, prioritizing accessibility for annotators with less linguistic expertise. The evaluation results indicate a balance between simplicity and depth, offering a practical resource for rapid syntactic analysis while maintaining high accuracy for basic syntactic relations in Arabic (Habash and Roth, 2009).

**CAMEL Treebank (CAMELTB)** is a comprehensive dependency treebank for both Modern Standard Arabic and Classical Arabic, annotated using guidelines aligned with CATiB. It includes approximately 188,000 words and 242,000 tokens from a variety of genres, including poetry, religious texts, and modern media. CAMELTB uses tools like CamelTools for tokenization and POS tagging, and the MALT parser for syntactic parsing. Its manual annotation process ensures high accuracy, with four native Arabic speakers involved in annotating and editing dependency relations. Evaluation results show the treebanks broad applicability across different Arabic dialects and registers, making it a valuable resource for linguistic research and NLP applications (Habash et al., 2022).

**Universal dependencies** for Arabic project utilizes dependency-based annotations from the Prague Arabic Dependency Treebank (PADT) and the Penn Arabic Treebank (NYUAD version) (Taji et al., 2017; Hajič et al., 2004). These datasets provide a robust foundation for analyzing Arabic syntax and morphology, addressing the challenges posed by the language's rich inflection and word formation. The annotations cover several layers, including part-of-speech tags, lemmas, morphological features, and syntactic relations. The project adopts a consistent approach to tokenization and morphological representation across different Arabic dialects, ensuring broad linguistic coverage. Evaluation of these treebanks emphasizes syntactic accuracy, with UD Arabic-PADT featuring 7,664 sentences and 242,056 tokens, and UD Arabic-NYUAD containing 19,738 sentences and 629,295 tokens. These treebanks offer comprehensive linguistic resources, enabling in-depth analysis of Arabic within the Universal Dependencies framework.

**AQMAR Arabic Wikipedia dependency tree corpus** (Habash et al., 2009) is derived from Arabic Wikipedia articles, annotated with part-of-speech (POS) tags and syntactic dependencies. This corpus comprises 1,262 sentences and 36,202 tokens, created with a semi-automated annotation process using the Brat annotation tool. The initial POS tagging was performed using the MADA system, followed by manual corrections. Dependency annotations were applied according to the CATiB Arabic dependency framework (Habash and Roth, 2009), ensuring high-quality syntactic representations. The dataset includes diverse topics, such as nuclear technology and football, providing valuable resources for semantic and syntactic analysis in various domains. While the annotations also cover named entities and semantic supersenses, the evaluation results primarily highlight improvements in syntactic parsing and dependency structure accuracy.

**ARL Arabic dependency Treebank**, developed by the US Army Research Laboratory (ARL) (Tratz, 2016), focuses on Arabic news and broadcast sources. This treebank is a restructured version of the Arabic Treebank (ATB) from the Linguistic Data Consortium, and it adopts a dependency grammar approach. Each sentence is analyzed based on a verb-centered structure, with other elements linked to the verb through directed relationships. The annotations include 11 columns, detailing the syntactic dependencies, POS tags, and lemmata, with each word or affix uniquely identified. Evaluation of the treebank involves measuring the quality of dependency relations and syntactic parsing, making it a crucial resource for Arabic language processing in military and defense applications. The dataset is available for further use in research and development of Arabic language technologies.

**OntoNotes 5.0** (Weischedel et al., 2013) is a large annotated corpus containing multiple linguistic layers, including syntactic, semantic, and discourse-level annotations. The Arabic portion, comprising 300K words, includes part-of-speech tagging, coreference, named entity recognition, and word sense disambiguation. The syntactic annotations use the Treebank framework, while the semantic annotations link word senses to an ontology. Evaluation results demonstrate high quality in both syntactic and semantic annotations, with comprehensive coverage of co-reference and named entities. The corpus provides a valuable resource for training machine learning models and evaluating Arabic language processing tasks. Available in both relational database format and text files, OntoNotes supports a range of research applications, including cross-linguistic studies and deep semantic parsing.

**I3rab Treebank** (Halabi et al., 2020) is a new Arabic dependency treebank that introduces innovative approaches to tokenization and dependency representation, focusing on the identification of primary words and the treatment of joined and implicit pronouns. The corpus is compared against a subset of the Prague Arabic Dependency Treebank (part-PADT), with evaluation results showing significant improvements in parsing performance. The I3rab dataset demonstrated a 7.5% increase in Unlabeled Attachment Score (UAS) and an 18.8% improvement in Labeled Attachment Score (LAS), highlighting the effectiveness of its unique approach. This treebank is intended to advance Arabic language processing by addressing gaps in previous dependency frameworks and offering a more accurate representation of syntactic relations in Arabic.

**Arabic Poetry Dependency Treebank (ArPoT)** (Al-Ghamdi et al., 2021) introduced ArPoT, the first dependency treebank specifically targeting classical Arabic poetry. The corpus consists of 2,685 verses (35,460 tokens) from 34 poets, annotated using the CATiB scheme, which is rooted in traditional Arabic grammar and supports future conversion to Universal Dependencies. ArPoTs annotation pipeline involved automatic parsing (using a tool trained on MSA) followed by extensive manual correction, with explicit attention to poetic-specific phenomena such as elision and cross-verse syntactic relations. Unlike most previous Arabic treebanks (e.g., Penn Arabic Treebank, CATiB, PADT) which are constructed for Modern Standard Arabic (MSA), ArPoT is dedicated to CA and captures its unique syntactic characteristics, making it a novel resource for the study of syntactic analysis in Arabic poetry.

**NArabizi Treebank** (Riabi et al., 2023) is a syntactically annotated corpus for North African Arabic (specifically Algerian dialect) written in Latin script—commonly known as NArabizi. The dataset consists of approximately 1,300 user-generated sentences, primarily sourced from online forums and song lyrics, with significant code-switching (36% French tokens). The latest version introduces major improvements, including standardized tokenization, corrections of morpho-syntactic and syntactic annotations following Universal Dependencies (UD) guidelines, and enhanced translation quality. Two new annotation layers were added: named entity recognition and offensive language detection, making the resource more versatile for downstream tasks. The treebank focuses exclusively on dialectal Arabic and does not include Modern Standard Arabic (MSA). However, its syntactic annotation—covering POS tags, morphological features, and dependency parses—serves as an essential benchmark for NLP tasks on noisy, low-resource Arabic varieties written in non-Arabic scripts. Experimental results showed that improving syntactic annotation quality led to significant gains in downstream dependency parsing and NER. The resource is freely available for research purposes.

**AraFast** (Alrayzah et al., 2024) is a large-scale, freely available Modern Standard Arabic (MSA) corpus aimed at addressing the shortage of comprehensive datasets for Arabic NLP research. The authors developed a multi-stage pipeline, combining automated and manual discovery of Arabic corpora from major repositories (such as GitHub, Kaggle, and Huggingface), followed by strict filtering for quality and genre, and extensive cleaning using custom algorithms. This process included deduplication, removal of noise, normalization, and segmentation with the WordPiece tokenizer. The final AraFast corpus comprises 112 GB of high-quality MSA and classical Arabic text from 48 different sources, reduced from an initial 833 GB of raw data through rigorous preprocessing. Importantly, it should be noted that AraFast is *not* a syntactically annotated resource such as a treebank; it does not include part-of-speech or syntactic structure annotations. Instead, AraFast provides a high-quality, segmented text corpus specifically designed for pretraining large transformer-based language models, using dynamic span-masking objectives. Both “base” (full corpus, 110M parameters) and “mini” (10GB) models were trained and evaluated. The experimental results showed that using segmented, clean data substantially improved model learning and stability (evidenced by lower training loss), while web-scraped noisy data led to training failures due to noise and data artifacts. While AraFast itself does not provide direct syntactic labels or parsing, its quality and scale make it a valuable foundational dataset. It indirectly supports advances in Arabic syntactic parsing by enabling the training of robust pre-trained language models, which can later be fine-tuned or adapted for downstream syntactic analysis tasks. Thus, AraFast serves as an important resource for both general and syntactic NLP applications in Arabic.

## 4 Available syntactic analyzers

Over the years, a wide array of Arabic syntactic analyzers have been developed, mirroring the progression of parsing techniques. Early parsers predominantly relied on manually crafted grammar rules and limited evaluation datasets, whereas subsequent systems leveraged machine learning trained on treebanks. In recent years, neural network and transformer-based parsers have achieved new state-of-the-art results by incorporating contextualized language models. The following subsections review representative Arabic parsers across these different paradigms, highlighting their approaches and reported performance.

### 4.1 Traditional syntactic analyzers for arabic

**Analyzer based on a recursive transition network** is a syntactic analyzer developed by Bataineh and Bataineh (2009) uses a Top-Down parsing approach based on Recursive Transition Networks (RTN), a concept derived from recursive transition grammars. The grammar for this parser is context-free, tailored to capture the most frequent sentence structures in Arabic. The approach applies both pattern-based rules and context-free rules, treating them as complementary. It was tested on 90 Arabic sentences, achieving an accuracy rate of 85.6%. However, the parser struggled with ungrammatical sentences and those outside the grammar's coverage, with 14.4% of sentences being unparseable.

**A'reb**, developed by Al-Daoud and Basata (2009), is a recursive, Top-Down parser designed to handle both lexical and syntactic analysis for Arabic sentences, focusing on verbal sentences. It utilizes recursive functions closely tied to production rules, allowing the parser's structure to reflect the grammar it interprets. Despite its functionality, the authors noted that further refinement is needed for complete effectiveness, with no quantitative evaluation results provided.

**Parse trees of Arabic sentences using NLTK** (Shatnawi and Belkhouche, 2012) is a rule-based approach utilizing Context-Free Grammar (CFG). The parser applies the NLTK recursive-descent algorithm to generate parsing trees for general and Quranic Arabic. Although it supports several NLP tasks, the authors pointed out that the model does not address more complex tasks like parsing dependencies, and no quantitative performance metrics were provided.

**Chart parser for analyzing Arabic sentences** (Al-Taani et al., 2012) is a Top-Down chart parser based on Context-Free Grammar (CFG) to analyze Arabic sentences. The parser's accuracy was evaluated on a small corpus of 70 sentences, with an average sentence length of 3.98 words, achieving 94.3% accuracy. However, the authors emphasized the need for further evaluation with a broader corpus to test the parser's reliability in diverse contexts.

**Context-free Grammar analysis top-down technique** (Al-qrainy et al., 2012) developed an Arabic parser based on Context-Free Grammar (CFG) and Top-Down recursive descent parsing using NLTK. The parser was tested on 150 Arabic sentences, achieving a high accuracy rate of 92% for verbal sentences and 98% for nominal sentences. However, the test set was small, and the types of sentences evaluated were unspecified, which limits the reliability of the results.

**ARSYPAR** (Khoufi et al., 2013) introduced an Arabic parser that uses supervised machine learning techniques, specifically Support Vector Machines (SVM). The parser was trained using features derived from the Arabic Treebank and focused on syntactic word classes. It was evaluated on a portion of the Arabic Treebank, achieving an F-score of 84.38%, demonstrating the efficacy of statistical methods in syntactic analysis.

**Industrial-strength parser** (Redjaimia et al., 2014) developed an advanced Arabic parser combining rule-based and statistical approaches to provide robust dependency and hierarchical constituent parsing. The parser underwent rigorous testing on a corpus of 300 Arabic sentences, achieving an F-score of 82%. This hybrid approach proved effective for applications like opinion mining in Arabic social media content, although the specific evaluation methodology was not detailed.

**Robust large-scale parser using AGFL formalism** (Ouersighni, 2014) used a rule-based approach with Affix Grammars over Finite Lattice (AGFL) formalism for parsing Arabic. The parser's robust performance was tested on 200 Arabic sentences, achieving a 95% success rate. However, it suffered from high ambiguity, with an average of 23.12 possible analyses per sentence, highlighting the trade-off between robustness and precision in this approach.

**Transducers parser** (Hammouda and Haddar, 2018) employed a transducers-based approach to parse Arabic nominal sentences. The system, which includes segmentation, preprocessing, and disambiguation phases, achieved a precision rate of 80% and a recall rate of 90% when tested on a corpus of 200 Arabic sentences. This method proved effective for nominal sentence parsing but may require further refinement for broader sentence structures.

**Inductive learning algorithm (ILA)** (Abu-Soud et al., 2018) developed an ILA to parse Arabic nominal and verbal sentences. The ILA generates parsing rules from a training dataset and achieved a 92.63% accuracy for previously unseen sentences. However, it performed better on verbal sentences compared to nominal ones, due to the structural complexity of the latter. The method demonstrated its potential for Arabic Natural Language Processing (ANLP) applications but highlighted the challenges of segmenting and tagging sentences accurately.

**Arabic parser based on CFG and classical grammar rules** (Ababou et al., 2017) proposed an Arabic parser using Context-Free Grammar (CFG) integrated with classical grammar rules. The system achieved 97% accuracy when tested on 200 nominal sentences, effectively identifying dependency relations. However, some verb tagging errors were noted, and the method's simplicity allows easy integration with other techniques, enhancing its adaptability in parsing Arabic sentences.

**Syntactic parsing using the NooJ linguistic platform** is syntactic analyzer employs a rule-based, linguistically driven approach for Arabic syntactic parsing (Bourahma et al., 2018). Focusing on enhancing lexicon classification, resolving ambiguities from morphological analysis, and modeling grammar based on nominal sentence structures. The evaluation of the system on 120 nominal sentences demonstrated a parsing accuracy of 95%, with disambiguation achieving an 86% accuracy. Despite the success, ambiguities remain in complex sentence structures, highlighting the challenge of fully capturing Arabics syntactic nuances. The approach proves effective in handling agglutination and word order variability.

**Multitask easy-first dependency parsing** uses a bottom-up parsing strategy with a multitask learning approach (Kankanampati et al., 2020). It simultaneously learns from two Arabic dependency treebanks (CATiB and UD) by parsing both syntactic and semantic features. Their model jointly parses sentences into both syntactic representations using shared and task-specific components, allowing partial parse trees in one formalism to inform decisions in the other. This approach is evaluated on parallel CATiB and UD treebanks—both automatically converted from parts 1–3 of the PATB—with standard train/dev/test splits. While these converted treebanks are not originally designed for dependency parsing, they are widely used as gold standards for syntactic analysis in Arabic NLP research. The multitask parser achieves substantial improvements over strong single-task baselines, with labeled attachment scores (LAS) of 86.15 for CATiB and 84.76 for UD, representing 9.9% and 6.1% error reductions respectively. The study highlights that explicit sharing of partial tree structures, rather than just neural parameter sharing, yields the largest gains, especially in complex syntactic constructions such as Idafa and modifiers.

**An Arabic probabilistic parser based on a property grammar** is a parser that uses a hybrid approach combining statistical modeling and rule-based parsing, based on a Property Grammar (PG) formalism (Bensalem et al., 2023). The parser applies a bottom-up parsing strategy using a Probabilistic Context-Free Grammar (PCFG) combined with a probabilistic Property Grammar (PPG). It integrates syntactic constraints and utilizes the CYK algorithm optimized with the Viterbi method. Evaluation on a test set of 400 sentences from ATB highlights the parser's ability to parse complex Arabic constructs with high precision. Compared to the Stanford parser (Dozat et al., 2017), it demonstrates better precision for specific linguistic phenomena, such as verbal sentences (88.3% vs. 81.9%) and nominal phrases (75.2% vs. 74.0%). However, it faces challenges in recall, particularly in capturing all relevant syntactic features.

**Bel-Arabi** combines both rule-based and learning-based approaches for Arabic syntactic parsing (Ibrahim et al., 2016). The system adopts a machine learning strategy for tasks like POS tagging and chunking, employing Conditional Random Fields (CRF) classifiers. The framework also integrates rule-based modules for grammatical marking, ensuring accurate syntactic analysis. With a high precision rate (90.44%) for analyzing 600 sentences, the system excels at identifying grammatical roles and diacritical marks. However, its performance declines when dealing with constructs like passive verbs, indicating areas for improvement, particularly in semantic analysis.

**Arabic parser using deep learning** employs deep learning techniques to tackle the complexities of Arabic syntax, utilizing bidirectional LSTM (BILSTM) models (Maalej et al., 2021). The system employs a statistical approach for syntactic parsing, utilizing deep learning models such as LSTM, GRU, and BILSTM, which are trained on word embeddings derived from the Penn Arabic Treebank (ATB). The BILSTM model demonstrated superior accuracy, achieving over 99% accuracy across various syntactic levels. The system effectively captures bidirectional contextual dependencies, making it a promising approach for Arabic syntactic parsing in NLP applications.

**Stanford Arabic parser** is a component of the Stanford CoreNLP suite that provides syntactic analysis of Arabic sentences using probabilistic context-free grammar (PCFG) models (Green and Manning, 2010). It is trained on the Penn Arabic Treebank (PATB) and operates in two main stages: first, it performs tokenization and segmentation—often using the Stanford Arabic Segmenter, and then applies syntactic parsing to produce hierarchical phrase structure trees.

The parser generates both constituency trees and part-of-speech (POS) tags, enabling deeper syntactic understanding necessary for downstream tasks like information extraction, question answering, and machine translation. It utilizes the CYK (Cocke–Younger–Kasami) parsing algorithm and supports features like n-best parses and probabilistic scoring, making it both powerful and flexible for diverse NLP applications. Although the parser itself doesn't perform sentiment analysis, its output supports sentiment models. Grammar-checking tools use the parser to identify and correct errors, and NER systems benefit from its contextual information. In educational settings, the parser teaches syntax and sentence structure, while businesses use it for text analytics, such as market research and customer feedback analysis. The parser's comprehensive applications demonstrate its versatility in understanding and processing natural language text.

The parser's performance on development test data for sentences under 40 words shows a factored F1 score (factF1) of 77.44% and dependency accuracy (factDA) of 84.05%. For the ATB part 3 Buckwalter grammar. These results highlight strong dependency parsing performance and suggest that inconsistencies in constituency annotations may account for the relatively lower F1 scores.

**Arabic tree adjoining grammar (ArabTAG V2.0)** or Arabic Tree Adjoining Grammar version 2.0, is an advanced syntactic and semantic analysis framework specifically designed for Modern Standard Arabic. Developed as part of a project led by researchers like Ben Khelil et al. (2023) and her collaborators, this grammar addresses the unique challenges posed by NLP, including its flexible word order, rich morphology, and the omission of diacritics in written texts. ArabTAG V2.0 builds on a prior manually defined grammar, enhancing it with an abstract representation called a meta-grammar. This abstraction allows linguists to describe both the syntax and semantics of Arabic more efficiently, facilitating the maintenance and expansion of the grammar. The framework includes 1,074 non-lexicalized syntactic rules and 27 semantic frames, focusing on predicate-argument structures.

The grammar is semi-automatically generated and is designed to cover a wide range of syntactical structures and linguistic phenomena. Experimental evaluations have shown that ArabTAG V2.0 can achieve a precision rate of 88.76% in syntactic analysis and about 95.63% in semantic analysis. This high level of accuracy demonstrates its capability to handle the complexity of Arabic syntax and semantics effectively.

**MASAQ parser** (Sawalha et al., 2025b) is a recent statistical parser developed for Classical Arabic, based on the newly released MASAQ dataset (Sawalha et al., 2025a). It applies supervised machine learning (Random Forest, LinearSVC, Logistic Regression) for fine-grained morphosyntactic analysis, focusing on dependency parsing in accordance with traditional Arabic *irab*. The MASAQ corpus includes 131,930 morphemes and 123,565 annotated syntactic functions over 77,408 Quranic words. Evaluation experiments report a best accuracy of 99.0% for syntactic role assignment using Random Forest, setting a new benchmark for Arabic syntactic analysis.

### 4.2 Modern neural and transformer-based approaches to arabic syntactic analysis

**Camel parser**, which includes versions 1.0 and 2.0 (Elshabrawy et al., 2023), integrates machine learning, specifically leveraging BERT-based embeddings for better contextual understanding, and applies biaffine attention mechanisms for dependency parsing. CamelParser 2.0 outperforms its predecessor by integrating advanced neural models, yielding improved parsing performance with a Labeled Attachment Score (LAS) of 91.3% and an Unlabeled Attachment Score (UAS) of 92.4%. The use of BERT and biaffine parsing results in a significant reduction in parsing errors, making it a robust tool for Arabic dependency parsing.

**Out-of-domain dependency parser** (Mokh et al., 2024) address the challenge of dependency parsing for Arabic dialects in an out-of-domain setting, given the lack of syntactically annotated dialectal corpora. Their approach uses a neural biaffine dependency parser (Dozat and Manning, 2016), trained on the Columbia Arabic Treebank (CATiB; Habash and Roth, 2009) and the Modern Standard Arabic (MSA) portion of the MADAR parallel corpus (Bouamor et al., 2018), and tested on a manually annotated set of Gulf, Levantine, Egyptian, and Maghrebi dialect sentences. They focus on the parsing of Idafa and coordination constructions, which are particularly challenging and structurally variable across dialects. The authors employ various domain adaptation strategies, including filtering training data by sentence length, removing sentential coordination, selecting structurally similar sentences based on POS bigram perplexity, and experimenting with different BERT-based embeddings. For in-domain evaluation, they used two syntactically annotated MSA datasets: CATiB and the MSA portion of the MADAR corpus, which consists of 2,000 sentences with full dependency. When trained and evaluated on CATiB, their parser achieved a Unlabeled Attachment Score (UAS) of 90.3% and a Labeled Attachment Score (LAS) of 88.7%. On the MADAR MSA dataset (2,000 annotated sentences), the parser reached a UAS of 97.9% and a LAS of 84.9%. However, performance drops significantly out-of-domain (e.g., UAS: 55.1–57.5%, LAS: 23.2–27.5% across dialects), but targeted adaptation techniques can raise LAS by up to 24 points for specific constructions. These results serve as an upper bound for parsing performance in MSA, given matched domain and annotation style.

**AraT5** (Nagoudi et al., 2022) is an Arabic text-to-text Transformer model trained on large-scale MSA and dialectal corpora, including AraNews (Nagoudi et al., 2020), El-Khair (El-Khair, 2016), and OSCAR (Suárez et al., 2020). While AraT5 does not function as an explicit syntactic analyzer, its sequence-to-sequence architecture and pretraining enable it to learn syntactic structures *implicitly*, as demonstrated by strong results on the ARGEN benchmark across seven tasks. AraT5 outperformed mT5 on 52 of 59 test splits, highlighting the effectiveness of implicit syntax modeling for Arabic language generation and understanding tasks.

**AraBERT** (Antoun et al., 2020) is a transformer-based language model specifically pre-trained for Arabic. Built on the BERT-base architecture (12 encoder layers, 768 hidden dimensions, 110M parameters), AraBERT introduces an Arabic-specific preprocessing pipeline by segmenting words into stems, prefixes, and suffixes using Farasa (Abdelali et al., 2016), followed by sub-word tokenization (SentencePiece, vocab size: 64K). The model is pre-trained on a large, diverse corpus comprising 70 million sentences (24GB) gathered from major Arabic news sources [notably the 1.5B Arabic Corpus (El-Khair, 2016) and OSIAN (Zeroual et al., 2019)], Modern Standard Arabic (MSA), and dialectal variants. Although AraBERT is not an explicit syntactic parser, its deep contextualized embeddings have shown strong performance on tasks highly dependent on syntactic and morphological understanding, making it widely adopted as a backbone for downstream syntactic analysis tasks. In evaluations across sentiment analysis, named entity recognition (NER), and question answering (QA), AraBERT consistently outperformed multilingual BERT and previous state-of-the-art models. The size and diversity of the training corpus and the Arabic-specific tokenization are key contributors to its robust syntactic modeling.

**MARBERT** (Abdul-Mageed et al., 2021) is a pre-trained deep bidirectional Transformer model specifically designed to address the diversity and informality of Arabic language varieties, especially on social media. Built on the BERT-base architecture (12 layers, 768 hidden units, 163M parameters), MARBERT is trained from scratch on a massive dataset of 1 billion Arabic tweets (128GB, 15.6B tokens), using a 100K WordPiece vocabulary. The pre-processing is intentionally minimal—removing only diacritics and normalizing URLs, usernames, and hashtags—to maximize the model's exposure to authentic, naturally occurring dialectal and noisy text. Importantly, while MARBERT is not a syntactic parser in the traditional sense, its deep contextualized representations have shown substantial impact on downstream tasks that depend on syntactic and morphosyntactic cues, such as named entity recognition, dialect identification, and question answering. For evaluation, MARBERT was assessed using the ARLUE benchmark (Abdul-Mageed et al., 2021), which consists of 42 diverse datasets across six task clusters (including tasks closely tied to syntactic analysis). MARBERT achieves state-of-the-art results on 37 out of 48 classification tasks, with an overall ARLUE macro-average score of 75.99, outperforming many larger multilingual models (such as XLM-RLarge, which is more than three times larger in parameters). Notably, MARBERT's strength is most pronounced in dialect identification and social meaning tasks—domains where syntactic variation is high and previous MSA-focused models struggled. To further address performance in tasks requiring longer context, the authors introduce MARBERTv2, which is obtained by continued pre-training of MARBERT on the same MSA data as ARBERT and the AraNews dataset, using a longer sequence length (512 tokens) for 40 additional epochs, resulting in exposure to 29 billion tokens.

**Dialect-specific pre-trained language models:** In addition to multidialect models like AraBERT and MARBERT, recent research has introduced several dialect-specific pre-trained language models, including CAMeLBERT (Inoue et al., 2021), SaudiBERT (Qarah, 2024b), and EgyBERT (Qarah, 2024a). CAMeLBERT comprises a suite of BERT-based models, each trained on a specific Arabic variant (Modern Standard Arabic, dialectal Arabic, or Classical Arabic), with pre-training corpora ranging up to 167GB and over 17 billion tokens. SaudiBERT is developed for the Saudi dialect using a corpus of 141 million Saudi tweets and forum data (totalling over 26GB), while EgyBERT targets the Egyptian dialect with more than 10GB of Egyptian tweets and forum texts. These models follow the BERT architecture and employ minimal pre-processing to preserve dialectal characteristics. Though not syntactic parsers, their contextualized representations significantly improve the performance of downstream tasks that require syntactic sensitivity.

Al-Ghamdi et al. (2023) proposed a novel approach for Arabic dependency parsing by fine-tuning BERT-based pre-trained language models, formulating the parsing task as a sequence labeling problem. Each token is assigned a composite label encoding both the head position and the dependency relation, and three head-encoding strategies (naive positional, relative positional, and relative POS-based) were systematically compared. The authors evaluated nine Arabic BERT-based models-including AraBERTv2, AraBERTv1, Camel-MSA, Camel-CA, ARBERT, and GigaBERT-on three treebanks: the Prague Arabic Dependency Treebank (PADT, Hajič et al., 2004), the Columbia Arabic Treebank (CATiB, Habash and Roth, 2009), and the Classical Arabic Poetry Dependency Treebank (ArPoT, Al-Ghamdi et al., 2021). Experimental results demonstrate that AraBERTv2 achieved the highest accuracy, reaching up to 84.03% UAS and 80.26% LAS on PADT, 87.54% UAS and 86.41% LAS on CATiB, and 79.79% UAS and 74.13% LAS on ArPoT. It should be noted that the work by Al-Ghamdi et al. (2023) does not propose a novel parser architecture, but rather adapts and thoroughly evaluates the sequence labelling approach using existing BERT-based pre-trained models for Arabic dependency parsing.

The provided Table 1 offers a comprehensive overview of Arabic syntactic analyzers, grouped primarily by their underlying methodologies: rule-based, hybrid, and neural approaches. Rule-based parsers, such as Recursive Transition Network (RTN), Chart Parser, AGFL Parser, and NooJ-based Analyzer, rely heavily on manually crafted grammatical rules and lexicons. These systems exhibit notable accuracy on controlled and limited sentence sets (85.6%–95%), yet they tend to struggle with linguistic coverage, robustness, and scalability to more complex or diverse texts. Hybrid approaches, including ARSYPAR, the Industrial-Strength Parser, Probabilistic Parser, and Bel-Arabi, integrate statistical or machine learning methods with linguistic rules. These parsers generally achieve intermediate levels of accuracy (82%–90%) and show enhanced robustness and broader linguistic coverage compared to purely rule-based methods. However, their performance is contingent upon annotated corpora and careful feature engineering, thus posing challenges in adaptability and maintenance. Neural network-based parsers, such as Camel Parser, AraBERT variants, and Deep-Learning Parsers utilizing transformer architectures, currently deliver state-of-the-art results (LAS and UAS typically ranging from 80% to over 90%). These models benefit significantly from extensive annotated corpora (PADT, CATiB, ATB) and demonstrate superior handling of Arabic morphology, syntactic ambiguity, and out-of-vocabulary words. Nonetheless, neural models require substantial computational resources and large annotated datasets, and they may face performance issues when encountering domain shifts or dialectal variations not represented in training data. Overall, these comparisons indicate that while early parsers laid important groundwork, the highest parsing accuracies for Arabic are currently achieved by transformer-based models and other recent neural approaches. While current parsers demonstrate substantial progress, future research directions include addressing domain and dialect adaptability, interpretability of neural models, and overcoming resource limitations through semi-supervised learning and multilingual transfer techniques. Such advancements will further bridge existing gaps and improve parser applicability across varied Arabic language scenarios.

**Table 1:** Comparative performance of Arabic syntactic analyzers.

**Analyzer**	**Approach followed**	**Evaluation results**	**Corpus size/name**
Recursive transition network	Top-down RTN; context-free + pattern rules	85.6 % accuracy	90 Arabic sentences
A'reb	Recursive top-down parser; production rules	–	Not specified
NLTK parser	Rule-based; CFG; recursive-descent	–	Not specified
Chart parser	Top-down chart parser; CFG	94.3% accuracy	70 Arabic sentences
CFG top-down	Recursive-descent CFG	92% verbal, 98% nominal accuracy	150 Arabic sentences
ARSYPAR	Supervised ML (SVM)	F-score 84.38%	Arabic Treebank subset
Industrial-strength parser	Hybrid (rule-based + statistical)	F-score 82%	300 Arabic sentences
AGFL parser	Rule-based; AGFL formalism	95% successful parses; high ambiguity	200 Arabic sentences
Transducers parser	Finite-state transducers; segmentation + disambiguation	Precision 80%, Recall 90%	200 Arabic sentences
Inductive learning algorithm	Rule induction from examples	92.63% accuracy	Unspecified (unseen sentences)
CFG + classical grammar	CFG plus traditional grammar rules	97% accuracy	200 nominal sentences
NooJ-based analyzer	Rule-based linguistic model	95% syntactic, 86% disambiguation accuracy	120 nominal sentences
Camel parser	BERT + biaffine dependency (ML)	UAS/LAS: 92.4/91.3	Not specified (likely ATB)
Multitask easy-first	Bottom-up, multitask learning	UAS/LAS: 88.08/86.15	CATiB Treebanks
Probabilistic parser	PCFG + property grammar, CYK	Precision 88.3% (verbal), 75.2% (nominal)	400 ATB sentences
Bel-Arabi	Hybrid ML (CRF) + rules	Precision 90.44%	600 sentences
Deep-learning parser	BiLSTM/LSTM/GRU	>99% accuracy	Penn Arabic Treebank
Stanford Arabic parser	PCFG + CYK	FactF1 77.44%, FactDA 84.05%	Penn Arabic Treebank
ArabTAG v2.0	Tree-adjoining grammar; meta-grammar	Precision 88.76% (syntax), 95.63% (semantics)	Not specified
MASAQ	Statistical parser (Random Forest)	Accuracy: 99.0%	MASAQ dataset: 123,565 syntactic functions
Camel-MSA	Fine-tuned BERT-based sequence labeling	UAS/LAS: 83.10/79.17	PADT: 282,384
Camel-MSA	Fine-tuned BERT-based sequence labeling	UAS/LAS: 86.47/85.29	CATiB: 169,319
AraBERTv1	Fine-tuned BERT-based sequence labeling	UAS/LAS: 82.76/78.82	PADT: 282,384
AraBERTv1	Fine-tuned BERT-based sequence labeling	UAS/LAS: 86.76/85.57	CATiB: 169,319
AraBERTv2	Fine-tuned BERT-based sequence labeling	UAS/LAS: 84.03/80.26	PADT: 282,384
AraBERTv2	Fine-tuned BERT-based sequence labeling	UAS/LAS: 87.54/86.41	CATiB: 169,319
ARBERT	Fine-tuned BERT-based sequence labeling	UAS/LAS: 80.37/76.11	PADT: 282,384
ARBERT	Fine-tuned BERT-based sequence labeling	UAS/LAS: 78.31/75.95	CATiB: 169,319
Arabic BERT	Fine-tuned BERT-based sequence labeling	UAS/LAS: 80.02/76.52	PADT: 282,384
Arabic BERT	Fine-tuned BERT-based sequence labeling	UAS/LAS: 82.65/80.59	CATiB: 169,319

## 5 Challenges in arabic syntactic analysis

Many of the difficulties in Arabic syntactic analysis are well-known, recent advances in machine learning, computational linguistics, and deep learning bring forth a new set of advanced challenges. These challenges not only stem from the traditional complexities of the language but also from the need to create sophisticated models capable of handling both contemporary and evolving linguistic phenomena. Below are some of the challenges that researchers are facing in Arabic syntactic analysis:

### 5.1 Unannotated domain-specific data and formalization gaps

While resources like the Penn Arabic Treebank (PATB) exist, they are heavily focused on formal texts and standard written Arabic, such as news articles. As more Arabic data comes from informal domains like social media, blogs, SMS, and chat conversations, syntactic structures in these domains become more difficult to annotate and generalize. These domains often contain non-standard spelling, abbreviations, and internet slang, and their syntax deviates from the rigid structures of MSA. Furthermore, Arabic-language syntactic structures in domain-specific applications (e.g., medical texts, legal documents, technical manuals) often require specialized syntactic theories and rules that current parsers are not equipped to handle. For example, the grammatical norms in technical writing might differ from colloquial speech, and handling these nuances requires more sophisticated annotation schemes that current treebanks and parsing models lack.

### 5.2 Ambiguities in syntactic structures due to ellipsis and zero pronouns

Arabic syntax features phenomena like ellipsis and zero pronouns that introduce ambiguity into sentence structure. These phenomena are particularly common in conversational Arabic and can result in incomplete syntactic structures that require contextual information to resolve. For instance, a sentence like “He went to the market, and she [went] to the store” in English uses an ellipsis, which may be straightforward to resolve in English, but in Arabic, this can be more complex due to the omission of verb phrases or pronouns without clear agreement. Zero pronouns, where the subject or object is omitted from a sentence because it can be inferred from context, add another layer of complexity. Accurately resolving these ellipses and zero pronouns in both MSA and dialectal varieties remains an unsolved challenge in syntactic parsing, particularly for systems that rely heavily on surface form rather than deeper contextual understanding.

### 5.3 Model generalization and domain adaptation

One of the most pressing challenges in Arabic syntactic analysis is the generalization of models across domains. While Arabic parsers have become quite effective for general text (e.g., news), they often fail when transferred to specific domains, such as healthcare, finance, or legal documents. Domain-specific vocabulary, sentence structures, and jargon can lead to significant degradation in performance when the models are not adapted properly. Traditional training methodologies that focus on general-purpose data are less effective for domain-specific tasks, and fine-tuning models for specialized domains remains an open area of research.

## 6 Conclusion and future directions

Arabic syntactic analysis has made significant strides over the past decade, transitioning from rule-based systems to more sophisticated machine learning and neural network models. Despite these advancements, several challenges remain, including handling dialectal variation, resolving ambiguities due to the lack of diacritics, and the need for larger, more diverse annotated datasets. As new systems and approaches are developed, the evaluation of Arabic syntactic analyzers will remain a critical challenge. Establishing more diverse and standardized benchmarks for evaluating Arabic parsers across dialects, genres, and domains is essential for guiding future improvements.

This paper systematically surveys and compares state-of-the-art methods for Arabic syntactic parsing, clearly highlighting the strengths and limitations of existing rule-based, statistical, machine learning, and hybrid approaches. It has also provided a comprehensive evaluation of essential resources, including prominent Arabic syntax treebanks. The comparative insights presented here serve as a foundational reference for researchers seeking to address the inherent complexities of Arabic NLP.

Future research should focus on leveraging advances in transformer-based models, such as multilingual and domain-adaptive language models, to enhance parser robustness across dialects and diverse textual domains. Joint models capable of simultaneously addressing morphological segmentation, POS tagging, and syntactic parsing should be developed to mitigate cascading errors. Additionally, increased efforts toward interpretability in neural systems and richer semantic annotations in Arabic Treebanks will significantly improve downstream NLP applications. Exploring cross-lingual transfer learning and semi-supervised learning techniques will be vital in overcoming current limitations related to the scarcity of annotated data, particularly for dialectal and low-resource Arabic varieties.

In conclusion, while significant progress has been made in Arabic syntactic analysis, ongoing challenges and evolving linguistic phenomena offer ample opportunities for further research. Advances in deep learning, multilingual modeling, and the expansion of dialectal resources are likely to drive the next wave of breakthroughs in the field.
